# Comparing children’s self-report instruments for health-related quality of life using the International Classification of Functioning, Disability and Health for Children and Youth (ICF-CY)

**DOI:** 10.1186/1477-7525-11-75

**Published:** 2013-05-04

**Authors:** Christina Petersson, Rune J Simeonsson, Karin Enskar, Karina Huus

**Affiliations:** 1 Jonkoping University, The Jonkoping Academy for improvement of health and welfare, Box 1026, Jonkoping, s-55111, Sweden; 2 CHILD Research Group, Jonkoping University, Jonkoping, Sweden; 3 University of North Carolina at Chapel Hill, School of Psychology and early Childhood Education, 105 G Peabody Hall, CB 3500, Chapel Hill, NC, 27599-3500, USA; 4 Department of Nursing Science, Jonkoping University, School of Health Science, Box 1026, Jonkoping, s-55111, Sweden

## Abstract

Children with chronic conditions often experience a long treatment which can be complex and negatively impacts the child's well-being. In planning treatment and interventions for children with chronic conditions, it is important to measure health-related quality of life (HrQoL). HrQoL instruments are considered to be a patient-reported outcome measure (PROM) and should be used in routine practice. Purpose: The aim of this study was to compare the content dimensions of HrQoL instruments for children's self-reports using the framework of ICF-CY. Method: The sample consist of six instruments for health-related quality of life for children 5 to 18 years of age, which was used in the Swedish national quality registries for children and adolescents with chronic conditions. The following instruments were included: CHQ-CF, DCGM-37, EQ-5D-Y, KIDSCREEN-52, Kid-KINDL and PedsQL 4.0. The framework of the ICF-CY was used as the basis for the comparison. Results: There were 290 meaningful concepts identified and linked to 88 categories in the classification ICF-CY with 29 categories of the component body functions, 48 categories of the component activities and participation and 11 categories of the component environmental factors. No concept were linked to the component body structures. The comparison revealed that the items in the HrQoL instruments corresponded primarily with the domains of activities and less with environmental factors. Conclusions: In conclusion, the results confirm that ICF-CY provide a good framework for content comparisons that evaluate similarities and differences to ICF-CY categories. The results of this study revealed the need for greater consensus of content across different HrQoL instruments. To obtain a detailed description of children's HrQoL, DCGM-37 and KIDSCREEN-52 may be appropriate instruments to use that can increase the understanding of young patients’ needs.

## Background

Health problems among children and adolescents have changed over the past century with an increased burden attributable to chronic conditions [1]. Children with chronic conditions often experience a long treatment which can be complex and negatively impact the child’s well-being. In planning treatment and interventions for children with chronic conditions it is important to measure quality of life and health-related quality of life (HrQoL) [2]. Quality of life can be described as a broad concept of a person’s well-being across different domains with health-related quality of life referenced as a sub domain of quality of life [3]. HrQoL instruments are considered to be a patient-reported outcome measure (PROM) and should be used in routine practice [4]. PROM can generally be defined as measures in which patients describe their experience of health, disease or treatment and can be used to assess generic dimensions such as well-being, health status or quality of life, as well as condition-specific activities and participation affected by a particular condition [5]. Identifying differences in perceived health-related quality of life among children with chronic health conditions [6] would enable health care professionals to focus on psychological domains of importance measured by different HrQoL instruments [7]. Recent reviews have been made of various HrQoL instruments for children and adolescents that refer to multidimensional aspects of health [8-11]. As the instruments vary in content related to health dimensions, a useful comparison can be made of HrQoL instruments using the framework and codes of the International Classification of Functioning, Disability and Health for Children and Youth (ICF-CY). ICF-CY has been used as a framework to compare HrQoL instruments in earlier studies [12-15] and has proved to be highly useful for analysis of content across measures. To clarify the content in patient reported measures for children with chronic conditions, a comparison of available instruments is needed to facilitate for health care personnel as well as researchers to select a patient-oriented instrument.

A chronic condition is characterized by its long duration and the fact that it is not amenable, but is managed across the life span. Modern medicine has made advances with potentially life-threatening chronic conditions so that children with conditions such as cancer or cystic fibrosis, are now surviving and living their lives. Chronic conditions can also vary to the extent where the treatment impacts children’s daily routines. Often it is not associated with acute problems but imposes restrictions on the child’s daily life [1], and can significantly influence the quality of the child’s development and life [16]. Young people with chronic conditions face social, psychological as well as physical challenges. They want to live a normal life like their friends, and these challenges become more important in the adolescent years [2]. With the increasing societal demand for better care and evaluation within health care there is a need for more comprehensive research on children’s health and measurement of children’s quality of life and health-related quality of life [17].

Health, quality of life or health-related quality of life are concepts which are increasingly assessed in health services as a complement to physical variables [10]. Health is a dynamic process and not a static state. A holistic perspective of health places focus on the person’s potential or capacity to actualize personal goals. WHO (World Health Organization) has defined health as a state of complete physical, social and mental well-being, not merely an absence of infirmity or disease. Many aspects of situations in life must be considered when discussing health in addition to age or gender [18]. The WHOQOL (World Health Organization Quality Of Life group) has defined quality of life as the person’s perception of his or her position in life (culture or value systems) in which he or she lives. This perception includes physical health, level of independence, psychological state, social relations and personal beliefs.

Quality of life thus refers to a person’s subjective judgment of their overall state within the cultural, environmental and social context [18]. Most individuals share common values in relation to quality of life, although priorities can vary by socio-demographic and personal characteristics [19]. Health-related quality of life is a multidimensional concept that refers to the subjective perception of physical, emotional, mental and social functioning and has its focus on the impact that health status has on quality of life [10,19,20]. Within this framework, there are two central aspects to be measured in HrQoL. The first aspect is the subjective dimension, meaning the person’s perspective. The second aspect is multidimensional including a broad range of factors, for example health status, treatment or disease, physiological, physical and social functioning [21]. HrQoL should also include an assessment of the patient’s satisfaction with treatment or outcomes [22]. Health and function should not be separated from other aspects of life and HrQoL should not be differentiated from the concept of quality of life. All aspects of life can affect health, and health and development are intertwined for all people [23].

The ICF-CY is an international taxonomy covering health aspects as well as health-related aspects of functioning and environment [24] and can be used as the basis for comparing instruments. ICF-CY has a bio-psychosocial approach of health, meaning different perspectives on health and are applicable both as a classification tool and a framework for research and clinical practice [25]. The system is built as a hierarchal system and includes four components with classification codes. Body functions (code b) refer to body systems and include psychological functions. Body structures (code s) represent anatomical parts of the body, such as organs and limb structures. Activities and participation (code d) cover the full range of life areas including basic learning, communication, interpersonal interaction as well as education. Components of contextual factors are the environmental (code e) and personal factors. The environmental factors consist of physical, attitudinal and social environment in the person’s life. Environmental factors have an impact on the other components of function and disability. Personal factors are identified under contextual factors, but they are not yet classified in ICF-CY [26]. The letters (b, s, d and e) refer to the components of the classification followed by a numeric code. This numeric code starts with the chapter number (first digit) followed by the second level (two digits). The third and fourth levels are one digit each.

### Measuring health-related quality of life

Quality of life and HrQoL instruments aim to systematically and scientifically record descriptions of health and illness of an individual. However, these instruments must be adapted for children, because children’s lives are different from those of adults [9]. Children should report their HrQoL whenever possible, but proxy versions of measures are available for parents to rate their children’s HrQoL as needed [10,27]. Only generic HrQoL instruments allow assessment of quality of life of both chronically ill children and healthy children. Measuring HrQoL in children and adolescents with chronic conditions may increase an understanding of their experiences or life situation [28]. HrQoL instruments reflect different theoretical bases and their content is designed to capture differences on a number of dimensions of health and functioning [8]. To examine broader psychosocial outcomes of illness and to provide a thorough understanding of the effects on children’s health status there is a need to analyze these instruments to determine the extent to which their content corresponds to experiences, activities and relevant contexts for children of different ages. The relationship between the social context and the children’s point of view is complex. Assessment of a child’s HrQoL must consider variables such as family functioning and relationships with friends. As the ICF version for children, (ICF-CY) [26] describes domains of developmental functioning and activities and environments relevant for children, for example family and school. The ICF-CY may be applicable for obtaining reliable and valid descriptions of child functional status and associated interventions and outcome measures [29]. The ICF-CY can facilitate documentation of functional aspects as the basis for promoting children’s participation and improving coordination of health services. Further, as a universal tool, the ICF-CY is appropriate to use to examine HrQoL instruments for children of different ages and across cultures [26,30]. If these instruments are constructed in culturally sensitive ways, they can be used in comparative studies across cultures. The aim of this study was to compare the content dimensions of HrQoL instruments for children’s self-reports using the framework of ICF-CY.

## Method

### Materials

For this comparative study, six HrQoL instruments were chosen for review (Table 1). Applicable instruments for inclusion were available in Swedish national quality registries for children with chronic conditions and had been translated to Swedish and tested for psychometric properties. The instruments were included if they were generic and designed for children 5 to 18 years of age. A generic instrument is designed to be applicable for the general population whereas condition-specific instruments are designed for one group with a specified illness, such as diabetes [3,31]. Condition-specific instruments were excluded in this study. Any instrument that measured health, quality of life and/or health-related quality of life was considered as a PROM and was chosen for this study. Only versions of children’s self-report were collected and if the instrument had more than one version, the long version was chosen. The resulting samples of instruments were: CHQ-CF (Child Health Questionnaire – Child Form), DCGM-37 (Disabkids Chronic Generic Measure −37 item), EQ-5D-Y (EuroQol Five Dimension – Youth version), KIDSCREEN-52, Kid-KINDL (Revised children Quality of Life Questionnaire), and PedsQL 4.0 (Pediatric Quality of Life Inventory TM).

**Table 1:** Summation of the instruments and the number of defined meaningful concepts coded to the ICF-CY

	**CHQ-CF**	**DCGM-37**	**EQ5D-Y**	**KIDSCREEN-52**	**Kid-KINDL**	**PedsQL 4.0**
Age	5-18	8-18	7-12	8-18	8-11	8-12
Dimensions	8	6	5	10	7	4
Items	87	37	6	52	30	23
Meaningful concepts	106	51	17	55	34	27
Body Functions*	29 (27%)	15 (29%)	4 (24%)	18 (33%)	15 (44%)	9 (33%)
Activities/participation*	58 (55%)	24 (47%)	11 (65%)	17 (31%)	8 (24%)	14 (52%)
Environmental factors*	5 (5%)	9 (18%)		13 (24%)	6 (18%)	3 (11%)
Defined as health condition*		1 (2%)		1 (2%)	1 (2%)	
Not definable/not covered*	1 (1%)			3 (5%)		
Not definable general health*	13 (12%)	1 (2%)	2 (11%)	3 (5%)	4 (12%)	1 (4%)
Not definable quality of life*		1 (2%)				

*Percent in parentheses counted within each instrument.

### Design and analytic procedure

This study used a comparative design to analyze the content of six HrQoL instruments. A review was made of every item in every instrument to determinate their linkage to codes in the ICF-CY classification (Swedish version) [32]. To compare the instruments, a deductive content analysis approach [33] was used and followed a set of sequential steps defined by Cieza et al. [34] as linking rules for coding with the ICF-CY. Before linking it is important to identify all meaningful concepts within each item. Response options are linked if they contain meaningful concepts and time interval is not linked to the ICF-CY. If an item contains more than one concept, each concept has to be linked separately [34]. Table 2 shows a summarization of the linking rules. The second and third level (when possible) in the ICF-CY chapters were chosen in the linking process.

**Table 2:** **Linking rules as described by Cieza et al.**[34]

**Rule nr**	**Description**	**Examples**
1	Acquiring knowledge of ICF-CY (chapters, domains and categories)	
2	Linking each meaningful concept to the most precise category	b28010 (Pain in head and neck)
3	Do not use the so-called “other specified” ICF categories, additional information shall be documented	
4	Do not use the “unspecified” ICF categories, use lower level of category	e4 (Attitudes)
5	designation not definable (nd), should be used when meaningful concept is not sufficient	If the concept refers to health, the designation should be nd- gh (not definable general health). If the concept refers to quality of life, the designation should be nd-qol (not definable quality of life)
6	If a meaningful concept is clearly a personal factor defined by ICF-CY, this can be documented with pf	pf (personal factor) gender, age
7	If there is no evidence of a meaningful concept and no personal factors are identified, then an assignment of nc	nc (not covered)
8	If a meaningful concept refers to health conditions or diagnosis it should be assigned hc	hc (Health condition) diabetes, asthma

### Reliability

To check for reliability of the linking process, two researchers were involved in the first step of the linking process. The first researcher (CP) coded the items in the selected instruments and discussed ambiguous items with a second researcher with experience in linking to ICF-CY. In the second step, ten percent of the coded items from each instrument were randomly sampled and coded separately by a third researcher to obtain inter-rater reliability. Reliability was deemed acceptable with 74 % agreement [32,35,36]. Differences in coding between raters did not involve linking items to ICF-CY categories, but rather if items were definable and could or could not be covered. For items not agreed upon, consensus was achieved by discussion between the first author (CP) and the second author (RS) and two other researchers with experience in coding to ICF-CY [32].

## Results

Of the 235 items in the six HrQoL instruments a total number of 290 meaningful concepts were identified and linked to ICF-CY codes. Overall, the concepts contained in the items of the instruments were linked to 88 different categories in ICF-CY (29 within body functions, 48 within activities and participation and 11 within environmental factors). No concepts were linked to the component body structures, and thirty-two meaningful concepts were not definable and additional codes were used (Table 1).

A review of individual instruments indicated that many of the items in the CHQ-CF were focused on physical daily functioning (chapter d4 and d5 in the ICF-CY). The categories representing participation (chapter d7 to d9) were not equivalent but varied with ten items coded as school education (d820) corresponding to school or school work. Environmental factors were only represented by five codes. Items on the DCGM-37 instrument corresponded to general tasks instead of specified daily functioning. Items related to participation were represented and well described, whereas environmental factors were referenced only by a few items concerning attitudes and a small percentage pertaining to medication (e1101). The EQ-5D-Y had a limited number of items; which describes dimensions rather than items. One item included several examples making it difficult to determine what the child would answer. Six ICF-CY codes were included within this particular example (d7500, d7601, d820, d880, d920 and d9201). The EQ-5D-Y did not include any environmental factors. KIDSCREEN-52 had items that represented daily activities and functions as well as items focused on participation and environmental factors. Both Kid-KINDL and KIDSCREEN-52 had several items that corresponded to environmental factors. However, neither had explicit items concerning daily activities and participation. PedsQL 4.0 differed from the other measures in that it had negatively stated items. This instrument covered daily activities of the child’s functioning, but included only one participation code from chapter d7 (interpersonal interactions and relationships). The number of environmental factors represented in PedsQL 4.0 was limited.

### Representation of body functions

The mental function codes, listed within chapter 1 of body functions, were referenced by all of the reviewed instruments (Table 3). Kid-KINDL had a detailed description of emotional functions and KIDSCREEN-52, DCGM-37 and PedsQL 4.0 also described range of emotions (b1522). Temperament and personality functions (b126) were found in every instrument with codes such as optimism (b 1265) and psychic stability (b1263). The code b180 (experience of self and time functions) occurred in all instruments except for EQ-5D-Y and PedsQL 4.0. Items that had been coded to this part of ICF-CY dealt with body image (b1801) and experience of self (b1800). There were four instruments referring to pain in some way: EQ-5D-Y referred to generalized pain (b2800) whereas CHQ-CF and Kid-KINDL referred specifically to pain in head and neck (b28010), and pain in the stomach and abdomen (b28012). The code for sleep functions (b134) was found only in three of the instruments (CHQ-CF, DCGM-37 and PedsQL 4.0). One item corresponded to stuttering, b330 (fluency and rhythm of speech functions) and was found in CHQ-CF.

**Table 3:** Frequencies showing how often body function categories were addressed in the reviewed instruments

**ICF-CY Categories**	**CHQ-CF**	**DCGM-37**	**EQ5D-Y**	**KIDSCREEN-52**	**Kid-KINDL**	**PedsQL 4.0**
b 110 Consciousness functions		1				
b 1252 Activity level		1		2	1	
b 1254 Persistence					1	
b 126 Temperament and personality functions	3	1		1		
b 1260 Extraversion	1					
b 1262 Conscientiousness	1					
b 1263 Psychic stability		1	1			1
b 1265 Optimism	2	2		3		
b 1266 Confidence	1			1		
b 1267 Trustworthiness	1					
b 130 Energy and drive functions					1	1
b 1300 Energy level		1				
b 134 Sleep functions	1	1				1
b 140 Attention functions	1			1	1	
b 1400 Sustaining attention		1				1
b 144 Memory functions		1				1
b 152 Emotional functions	11	3	2	2	2	2
b 1521 Regulations of emotion					1	
b 1522 Range of emotion		1		3	1	1
b 1602 Content of thoughts					1	
b 180 Experience of self and time functions	1			1	2	
b 1800 Experience of self		1			2	
b 1801 Body image	2			2		
b 1802 Experience of time				2		
b 280 Sensation of pain	2					1
b 2800 Generalized pain			1			
b 28010 Pain in head and neck	1				1	
b 28012 Pain in stomach and abdomen					1	
b 330 Fluency and rhythm of speech functions	1					

### Representation of activities and participation

Codes for general tasks and demands (chapter d2 in the ICF-CY) were found in all instruments, but in different ways (Table 4). DCGM-37, for example, included undertaking multiple tasks, handling stress (d 220) and other psychological demands (d240). This code was also represented in KIDSCREEN-52 and Kid-KINDL. Chapter four (mobility) and chapter five (self-care) in the ICF-CY cover moving or changing body positions and caring for oneself, such as washing, dressing or eating. The CHQ-CF was the instrument with the most items related to codes in these chapters. Basic interpersonal interactions (d710) were covered in all of the instruments, except for EQ-5D-Y. This code was assigned to five items in the CHQ-CF, with one of the items dealing with stealing. The instruments with the most frequent codes in the area of interpersonal interactions and relationships, were the CHQ-CF and DCGM-37. Chapter d8 in the ICF-CY covers major life areas and all of the instruments had items to which school education (d280) was assigned. Items related to codes for engagement in play (d880) and shared cooperative play (d8803) were found in DCGM-37, Kid-KINDL and PedsQL 4.0. Items related to codes for community, social and civic life (last ICF-CY chapter), were not found in Kid-KINDL, but recreation and leisure (d920) and sports (d9201) were found in EQ-5D-Y, and socializing (d9205) was found in the remaining instruments.

**Table 4:** Frequencies showing how often activities and participation categories were addressed in the instruments

**ICF-CY Categories**	**CHQ-CF**	**DCGM-37**	**EQ-5D-Y**	**KIDSCREEN-52**	**Kid-KINDL**	**PedsQL 4.0**
d 155 Acquiring skills		1			1	
d 220 Undertaking multiple tasks		1				
d 2200 Carrying out multiple tasks						1
d 2202 Undertaking multiple tasks independently		1				
d 230 Carrying out daily routine			1			
d 240 Handling stress and other psychologicaldemands		2		1	1	
d 250 Managing one’s own behavior	3					
d 2503 Acting predictably		1				
d 2504 Adapting activity level	1					
d 330 Speaking	1	2				
d 410 Changing basic body positions	1					
d 4105 Bending	1					
d 4300 Lifting	1					1
d 435 Moving objects with lower extremities	1					
d 450 Walking	1		1			
d 4500 Walking short distances	1					1
d 455 Moving around	6		1	1		
d 4551 Climbing	1	1				
d 4552 Running	1	1		2		1
d 4553 Jumping	1					
d 4554 Swimming				1		
d 465 Moving around using equipment	1			1		
d 510 Washing oneself	1		1			
d 5101 Washing whole body						1
d 530 Toileting	1					
d 540 Dressing			1			
d 5400 Putting on clothes	1					
d 550 Eating	1					
d 570 Looking after one’s health	2	2			1	1
d 5702 Maintaining owns health	2	2				
d 6406 Helping to do housework	1					1
d 710 Basic interpersonal interactions	5	1		2	1	1
d 7101 Appreciation in relationships		1				
d 7102 Tolerance in relationships		1				
d 720 Complex interpersonal interactions		1				
d 750 Informal social relationships	1					
d 7500 Informal relationships with friends			1			
d 760 Family relationships	1					
d 7601 Child–parent relationships			1	1		
d 820 School education	10	1	1	4	2	3
d 8201 Maintaining educational program				1		
d 8202 Progressing in educational program					1	1
d 880 Engagement in play		1	1			
d 8803 Shared cooperative play		1			1	1
d 9103 Informal community life		1				
d 920 Recreation and leisure		2	1	2		
d 9201 Sports	2		1			1
d 9205 Socializing	9			1		

### Representation of environmental factors

The review of the HrQoL instruments revealed that there were no items in the EQ-5D-Y with reference to the representation of environmental factors (Table 5). Items concerning drugs (e1101) were found in DCGM-37, whereas financial asset (e1650) was used in KIDSCREEN-52 with reference to money for expenditures and money to do things with others. The most common codes for environmental factors in the instruments related to support and relationships for example the codes immediate family (e310) and friends (e320) occurring in half the instruments. KIDSCREEN-52 was the single instrument with the most items pertaining to environmental factors. CHQ-CF included two items related to attitudes (chapter d4) and the code for individual attitudes of immediate family members (e410) was found in KIDSCREEN-52, Kid-KINDL and PedsQL 4.0. Reference to individual attitudes of acquaintances, peers, colleagues, neighbors and community members (e425) occurred in all of the instruments, except for PedsQL 4.0. DCGM-37 had one item that dealt with how to be treated differently by teachers, which was assigned to individual attitudes of other professionals (e455).

**Table 5:** Frequencies showing how often environmental factors were addressed in the instruments

**ICF-CY Categories**	**CHQ-CF**	**DCGM-37**	**EQ-5D-Y**	**KIDSCREEN-52**	**Kid-KINDL**	**PedsQL 4.0**
e 1101 Drugs		6				
e 1650 Financial assets				3		
e 310 Immediate Family	1			2	3	
e 320 Friends				3	1	1
e 325 Acquaintances, peers, colleagues, neighbors,and community members	2					
e 360 Other professionals				1		
e 4 Attitudes	1					
e 410 Individual attitudes of immediate familymembers				2	1	1
e 420 Individual attitudes of friends						1
e 425 Individual attitudes of friends, acquaintances, neighbors,peers, colleagues and community members	1	2		2	1	
e 455 Individual attitudes of other professionals		1				

The ICF-CY framework extends beyond physical dimensions and provides information on the limitation of content of HrQoL instruments. Linking items to ICF-CY codes revealed the heterogeneity of the instruments. The differentiation between activity (chapter d2-d6 in ICF-CY) and participation (chapter d7 –d9 in ICF-CY) was not made, but the emphasis is on categories regarding activity rather than participation as the instruments include items that generally correspond to physical activities. Also, categories for environmental factors were relatively limited and highly variable in terms of codes covered.

## Discussion

The ICF-CY proved to be useful in this study for comparing HrQoL instruments and revealing the heterogeneity of these instruments regarding the coverage of body functions, activities and participation, and environmental factors. The diversity of the compared instruments becomes clear when content in the HrQoL instruments was coded to the framework of ICF-CY. On an overall basis, the frequency of items to which codes could be assigned across measures was 132 for activities and participation, 90 for body functions and 36 for environmental factors (Table 1). It should be noted that one instrument, the CHQ-CF, accounted for 55 % of the coded items in activities and participation and 27 % of the coded items in body functions. Similarly, 24 % of items coded for environmental factors came from a single instrument, KIDSCREEN-52, which points out the differences between instruments. The comparative approach provides insights into the differences with respect to the breadth of the dimensions measured, and the precision of measures, which has been established in earlier studies [12,15,37].

The comparison of the HrQoL instruments based on the ICF-CY provides information that can help health care professionals to select a suitable instrument to measure HrQoL. The selection of instruments should be made depending on aspects of the child’s functioning, activities and participation that are of importance to study. Over time, the use of generic instruments in national quality registries can facilitate the comparison of HrQoL of children with chronic health conditions. This study reveals the focus each instrument has on function, activity and participation, and environmental factors from an ICF-CY perspective. The ability to identify the utility of specific pediatric HrQoL instruments is important when identifying children with the greatest needs and to target interventions for those children [38]. A single instrument with a smaller number of items can only provide a general description of HrQoL. To obtain a more detailed description of HrQoL in quality registries, two or more instruments should be chosen, for example DCGM-37, which covers activities and participation combined with KIDSCREEN-52, which covers body functions, this to gain a broader perspective of the child’s HrQoL.

Depending on the purpose of the intended use, consideration of several questions should guide the selection of an HrQoL instrument. The first question should seek to determine whether the instrument is valid for the target population. Second, does the content in the instrument correspond to the information of interest for collection? In addition to questions regarding brevity and ease of administration of instruments, it is also important to consider interpretability of results obtained using HrQoL instruments. The instruments should demonstrate validity and reliability as well as sensitivity, if the aim is to monitor health and quality of life over time [8]. Children undergo rapid development and maturation from childhood to adulthood with corresponding changes in life over time, making the measurement of health, quality of life and HrQoL a challenging task [6]. From a developmental standpoint, consideration needs to be given to the fact that children are continually changing [39] which influences the extent to which they understand the questions being asked and how they experience their condition. The above issues reflect the challenges of using HrQoL instruments and are important in selecting patient reported measures to complement other measurements for ensuring high quality in health care. The benefits of using HrQoL instruments for children with chronic conditions are based on the extent to which they enhance collaboration among researchers and health care professionals. Further, it is important that children are consulted on their treatment goals by health care professionals [40]. Concerning items in the instruments, the wording should be taken into consideration, for example the use of a negative language [41], as well as number of items. The balance between a large number of items and the child’s risk of failing to answer has to be considered when selecting the instrument. Further, an important question is how the assessment of HrQoL and its determinants contributes to the understanding of children’s needs and to the ability to provide improved care [20]. Therefore, the continued development of HrQoL instruments for children with chronic conditions is of increasing importance to enable optimal care for children with chronic conditions.

### Methodological considerations

The linking rules [34] were used for coding items from the HrQoL instruments to the ICF-CY. However, in the absence of a detailed definition of coding meaningful concepts, every item in the instruments had to be read in its context. When linking items, it is obvious that some level of interpretation had to be accepted, recognizing the possibility of the researcher’s perception or orientation. Several ICF-CY categories were general in nature and open to consideration in terms of meaningful concepts, a finding reported by others [15,42]. Although the extent of coverage was varied, the content of the HrQoL instruments was represented by domains and codes of the ICF-CY. As described by other researchers, comparison of HrQoL instruments using the ICF-CY can demonstrate the nature and scope of their content. As many instruments lack a defined focus for effective decision-making, this comparison have revealed the uniqueness of each instrument [12,15]. The compared instruments were translated to Swedish, but do exist in several countries and have been developed cross-nationally and have been tested for psychometric properties. The psychometric properties are not discussed here, but can be found in other reviews [8,43-45].

### Limitations

There are several limitations of this study. The instruments compared in this study are measuring either health, quality of life or HrQoL, which makes the comparison complicated. Further, the instruments assess different dimensions and the numbers of items vary across instruments from 6 to 87 items. The linkage revealed that categories were addressed differently in the instruments, suggesting difficulty in using the ICF-CY to distinguish some categories. For example the category emotional functions (b 152) was found to correspond to sadness, anxiety, happiness or anger, which points out the limitations of ICF-CY and the need to develop the classification. A final limitation is that, the instruments reviewed in this study only refer to child reports and do not included proxy-reports of HrQoL.

### Conclusions and clinical implications

The comparison of HrQoL instruments in this study may provide health care professionals and the national quality registries with insights when selecting instruments for assessing HrQoL of children with chronic conditions. The results of this study revealed the need for greater consensus of content across different instruments as they differ in focus on ICF-CY categories. To obtain a detailed description of HrQoL in quality registries, DCGM-37 and KIDSCREEN-52 may be appropriate instruments to use. Further research is needed for the development of instruments that are user-friendly and computer-based, which may facilitate their expanded use prior to the health care visit. The use of patient reported measures can contribute a positive influence on health status. It can increase the understanding of patients’ needs and address the issues that are important from the patient’s point of view [4]. Health care professionals should be encouraged to think beyond conventional routines, especially concerning children with chronic conditions. The use of HrQoL instruments in health care can make a significant contribution in facilitating priorities and goals shared by children and their health care providers. In conclusion, the results confirm that ICF-CY provides a good framework for content comparisons that evaluate similarities and differences of ICF-CY categories.

## Abbreviations

ICF-CY: International Classification of Functioning, Disability and Health for Children and Youth; HrQoL: Health-related Quality of Life; PROM: Patient Reported Outcome measures.

## Competing interests

The authors declare that they have no competing interests.

## Authors’ contributions

CP, as the first author, was responsible for the linking procedure and is primarily responsible for outcome assessments and writing the manuscript. KE had the responsibility for the study design, supervised the execution of the study, and the manuscript writing. RS participated in the linking procedure and drafting the manuscript. KH had the primary responsibility for the study design, supervised the execution of the study and drafting the manuscript. All authors read and approved the final manuscript.

## Authors’ information

All authors’ are members of CHILD Research Group, School of Health and Welfare, School of Health Science, Jonkoping University. CHILD is an acronym for Children, Health, Intervention, Learning and Development and the primary research focus is on interventions, participation, learning, health and everyday functioning in children with special needs.

RS is Professor of School of Psychology and Early Childhood Education and fellow at the Frank Porter Graham Child Development Institute, University of North Carolina at Chapel Hill, USA.
